# Dietary Linoleic Acid Elevates the Endocannabinoids 2-AG and Anandamide and Promotes Weight Gain in Mice Fed a Low Fat Diet

**DOI:** 10.1007/s11745-013-3842-y

**Published:** 2013-10-01

**Authors:** Anita Røyneberg Alvheim, Bente E. Torstensen, Yu Hong Lin, Haldis Haukås Lillefosse, Erik-Jan Lock, Lise Madsen, Livar Frøyland, Joseph R. Hibbeln, Marian Kjellevold Malde

**Affiliations:** 1 National Institute of Nutrition and Seafood Research (NIFES), P. O. Box 2029, Nordnes, 5817 Bergen, Norway; 2 Department of Biomedicine, University of Bergen, Bergen, Norway; 3 National Institute on Alcohol Abuse and Alcoholism, 5625 Fishers Lane, Rockville, MD 20852 USA; 4 Department of Biology, University of Copenhagen, Copenhagen, Denmark

**Keywords:** Endocannabinoids, Linoleic acid, PUFA, n-6, n-3, Low fat, Obesity

## Abstract

Dietary intake of linoleic acid (LNA, 18:2n-6) has increased dramatically during the 20th century and is associated with greater prevalence of obesity. The endocannabinoid system is involved in regulation of energy balance and a sustained hyperactivity of the endocannabinoid system may contribute to obesity. Arachidonic acid (ARA, 20:4n-6) is the precursor for 2-AG and anandamide (AEA), and we sought to determine if low fat diets (LFD) could be made obesogenic by increasing the endocannabinoid precursor pool of ARA, causing excessive endocannabinoid signaling leading to weight gain and a metabolic profile associated with obesity. Mice (C57BL/6j, 6 weeks of age) were fed 1 en% LNA and 8 en% LNA in low fat (12.5 en%) and medium fat diets (MFD, 35 en%) for 16 weeks. We found that increasing dietary LNA from 1 to 8 en% in LFD and MFD significantly increased ARA in phospholipids (ARA–PL), elevated 2-AG and AEA in liver, elevated plasma leptin, and resulted in larger adipocytes and more macrophage infiltration in adipose tissue. In LFD, dietary LNA of 8 en% increased feed efficiency and caused greater weight gain than in an isocaloric reduction to 1 en% LNA. Increasing dietary LNA from 1 to 8 en% elevates liver endocannabinoid levels and increases the risk of developing obesity. Thus a high dietary content of LNA (8 en%) increases the adipogenic properties of a low fat diet.

## Introduction

The endocannabinoid signaling system includes the endogenous ligands 2-arachidonoylglycerol (2-AG) and *N*-arachidonoylethanolamine (anandamide or AEA) [1–4] and the cannabinoid receptors (CB_1_ and CB_2_) [5, 6]. Genetic and pharmacologic impairment of the CB_1_ receptor have demonstrated the role of the endocannabinoid system in energy homeostasis by inhibiting food intake and reducing body weight, both occurring independent of energy intake [7–9]. Activation of the cannabinoid receptor CB_1_ by endocannabinoids or exogenous agonists, centrally and peripherally, favors metabolic processes that stimulate appetite, increase food intake, activates fat storage pathways and down regulates catabolism resulting in adipose accretion [7, 10]. In the liver, CB_1_ activation increases *de novo* lipogenesis through stimulation of fatty acid synthase activity leading to fatty liver and obesity [10]. Under normal circumstances the endocannabinoid system is only activated on demand in response to acute stimulation, but has been found to be tonically overactivated in animal models of genetic and diet-induced obesity [10–15]. Down regulation of excessive endocannabinoid system activity is being actively pursued to reduce obesity [16]. Because pharmacological blockade of the CB_1_ receptor is effective in treating obesity and related metabolic derangements [17, 18], a dietary approach to diminish endocannabinoid hyperactivity may represent a safer alternative than pharmaceuticals [19, 20]. In this study, we are addressing an underlying cause of endocannabinoid overactivation that can potentially be translated into a safe alternative to prevent human obesity.

Current dietary guidelines recommend the replacement of saturated fat with polyunsaturated fat to reduce the incidence of cardiovascular disease [21, 22], and have increased the consumption of vegetable oils. The estimated per capita consumption of soybean oil, one of the major dietary sources of LNA in the US, has increased more than 1,000-fold from 1909 to 1999, increasing the availability of LNA from 2.8 to 7.2 en% [23]. We previously reported a robust positive correlation between the prevalence of obesity in the US and soybean oil, and with the other primary sources of LNA such as poultry and shortening [14]. Thus we believe that the high intake of LNA, of which soybean oil is the greatest contributor in the US diet, is a strong contributor to the obesity epidemic.

The endocannabinoids are endogenous lipid mediators formed from the pool of 20 carbon n-6 fatty acids present in membrane phospholipids (PL) [24]. The n-6 fatty acid arachidonic acid (ARA, 20:4n-6) in phospholipids is the precursors of the two best characterized endocannabinoids 2-AG and AEA. Since n-3 and n-6 fatty acids cannot be synthesized *de novo* the fatty acid composition in tissues is reflected by the dietary intake of these fatty acids [25]. An epidemiological report linked increasing intake of LNA over time to increased prevalence of obesity and postulated that ARA-induced elevation in 2-AG may have altered the energy balance towards obesity [26]. In a recent study we modeled the increase in human consumption of LNA and demonstrated that increasing dietary levels of LNA from 1 to 8 en%, elevated the levels of ARA–PL, 2-AG and AEA in liver and promoted obesity in mice fed high fat diets of 35 and 60 en% fat [14]. We showed that it was the n-3 and n-6 fat composition of the diets, rather than the total amount of fat that determined the obesogenic properties of the diets [14]. In the same study, we also reversed the obesogenic effect of high fat diets by isocalorically decreasing dietary LNA from 8 to 1 en% and attenuated the ARA-dependent excessive endocannabinoid activity [14]. Furthermore, we demonstrated that reducing the ARA–PL precursor pool by adding 1 en% eicosapentaenoic acid (EPA, 20:5n-3) and docosahexaenoic acid (DHA, 22:6n-3) to 8 en% LNA diets reversed both stimulation of endocannabinoid activity and the obesogenic effect of high fat diets [14]. Thus, the obesogenic potential of high fat diets can be reversed by selectively reducing the essential fatty acid precursors of endocannabinoids available only from dietary sources. As we have previously shown that 8 en% LNA elevate endocannabinoid levels and induce adiposity in high fat diets, we sought to investigate whether a low fat diet could be made obesogenic by similar elevations of dietary endocannabinoid precursors. Therefore we aimed to determine if selective isocaloric inclusion of 8 en% LNA in a low fat diet (12.5 en%) and medium fat diet (35 en%) resulted in endocannabinoid hyperactivity and obesity.

## Materials and Methods

### Ethical Statement

All animal experiments were approved by the National Animal Health Authorities (Norwegian approval identification 1973). Care and handling were in accordance with local institutional recommendations and rules, and no adverse events were observed. The animals were anesthetized with isoflurane to minimize suffering before decapitation.

### Animals

Male mice (*n* = 36), 6 weeks of age (C57BL/6j, Taconic, Denmark) were randomly assigned to four experimental diets (*n* = 9) (Table 1) and housed individually. The animals were maintained on a 12:12 h light–dark cycle at 29 ± 1 °C. All animals were sacrificed at 22 weeks of age.

**Table 1:** Diet composition and fatty acid profile of diets

	Low fat diets (12.5 en%)		Medium fat diets (35 en%)	
	1 en% LNA	8 en% LNA	1 en% LNA	8 en% LNA
g/kg				
Cocosa (coconut oil)	3	–	129	85
Safflower oil	1	44	–	42
Olive oil	44	–	33	31
Flaxseed oil	2	6	3	7
Total oil added (g/kg)	50	50	165	165
% energy derived from				
Fat	11	11	32	34
Carbohydrate	71	71	45	44
Protein	18	18	23	22
(% of energy)				
Sum SFA	2.5	1.5	25	17
Sum MUFA	9	2	7	8
18:2n-6	1	8	1	8
20:4n-6	–	–	–	–
Sum n-6	1	8	1	8
18:3n-3	0.3	0.9	0.4	1
20:5n-3	–	–	–	–
22:6n-3	–	–	–	–
Sum n-3	0.3	0.9	0.3	1
Total n-6/n-3 ratio	3	9	3	8
Estimated n-6 HUFA^1^ (%)	56	72	53	72
Estimated Omega-3 Index^2^	11	5	11.5	5.5

All diets were supplemented with 60 mg/k ethoxyquin, 2.5 g/kg choline bitartrate, 3 g/kg l-cystein, 13 g/kg vitamin mix, 47 g/kg mineral mix, 50 g/kg sucrose, 50 g/kg cellulose, 100 g/kg corn starch. Energy (kcal) of diets; LF diets; 3,900, MF diets; 4,500

*LNA* linoleic acid, *SFA* saturated fatty acids, *MUFA* monounsaturated fatty acids, *HUFA* highly unsaturated fatty acids (>20 carbon)

^1^ Calculated from the Lands equation [25], n-3 HUFA = 100 − n-6 HUFA

^2^ Omega-3 index: (omega-3 HUFA × 0.32) − 3.5 [28, 59]

### Water and Food

Food provided as pellets was available ad libitum for 16 weeks. The diets contained 12.5 en% fat (LFD) and 35 en% fat (MFD) and 18 and 22 en% protein from casein (LFD and MFD respectively). Dextrin was used as a neutral source of carbohydrate, and was used to compensate for the lower fat content in the LFDs. In order to model the increase in human consumption of LNA from 1 to 8 en% and isolate LNA and ALA as controlled variables different oils were mixed to obtain the specific fatty acid profile of the diets listed in Table 1. Food intake was measured every other day by weighing each food cup and spillage and subtracting the previously collected weight. Body weight was recorded once a week for all animals.

### Endocannabinoids

Mice were sacrificed by decapitation and brain and liver were quickly snap-frozen in liquid nitrogen. The 2-AG and AEA were extracted and determined by gas chromatography/mass spectrometry/mass spectrometry (GC/MS/MS) as previously described [14].

### Fatty Acid Profile

Liver, red blood cells (RBC) and adipose tissue lipids were extracted with chloroform: methanol (2:1 v/v) and phospholipids were separated from neutral lipids by solid phase extraction (SPE). Epididymal adipose tissue (eWAT) and liver lipids were evaporated to dryness and recovered in chloroform to a concentration of 50 mg/mL lipids. An aliquot of 200 μL (10 mg lipids) was applied to a SPE column (Isolute). RBC lipids were evaporated to dryness and recovered with three washings of 100 mL chloroform and deposited to the SPE column. Neutral lipids were eluted with 10 mL chloroform/methanol (98:2 v/v) and polar lipids were eluted with 20 mL methanol. The fatty acid composition in the phospholipid fraction of liver and RBC, and the neutral fraction of adipose tissue were analyzed by GC as previously described [27].

### Blood Chemistry

Blood was collected in an Eppendorf tube from the bleeding carcass after decapitation and separated into erythrocytes and plasma. Plasma hormone levels were determined using commercially available ELISA kits in accordance with manufacturer’s instructions for insulin (DRG Diagnostics, Ultrasensitive ELISA, mouse, detection limit 0.025 μg/L, coefficient of variation within assay ~2 %, between assay ~4 %), leptin (ALPCO Immunoassays, Leptin (Mouse/Rat) ELISA, range 15–1,600 pg/mL, sensitivity 10 pg/mL, inter-assay and intra-assay variation coefficients <4.7 and <4.4 %, respectively) and adiponectin (ALPCO Immunoassays (Mouse) Total, HMW ELISA, range 0.125–8 ng/mL, sensitivity 0.032 ng/mL, inter-assay and intra-assay coefficient of variation ~3.5 %.).

### Histology

Sections of adipose tissue were fixed in 4 % formaldehyde in 0,1 M phosphate buffer (PB) for 24 h, washed in PB, dehydrated in ethanol, and embedded in paraffin after clearing with xylene. 5 μm thick sections of the embedded tissue were stained with eosin and hematoxylin. Sections were visually examined using an Olympus BX 51 binocular microscope (Tokyo, Japan) fitted with a Nikon DS-Fi1 camera (Digital Sight DS-Fi1, Nikon, Japan). Adipocyte size was measured using the interactive measurement module of an image analysis system equipped with an Olympus microscope, a Nikon DS-Fi1 camera and NIS-elements software (Nikon, Japan). Two hundred adipocytes per tissue (*n* = 2) were randomly selected and their size was measured by drawing a horizontal line between the cell membranes.

### Immunohistochemistry

Samples were processed in formaldehyde as described above. Then 5-μm sections were deparaffinized, rehydrated, and endogenous peroxide was inactivated (3 % hydrogen peroxide). To reduce non-specific staining the sections were incubated in heat-inactivated normal goat serum (10 %, 10 min). Sections were then incubated overnight at 4 °C with rat anti-mouse F4/80 (1:500; Serotec, Germany), subsequently washed in tris buffered saline (TBS, 3 times, 10 min) and incubated with HRP-conjugated goat anti-rat IgG (1:250; Serotec, Germany) for 2 h. After washing in TBS (3 × 10 min) specific binding was visualized using diamino benzidine. The immunohistochemistry was performed and examined by a scientist masked to the experimental conditions.

### Statistics

All data are analyzed using StatSoft, Inc. (2009) STATISTICA (data analysis software system), version 9.0. Data were analyzed for homogeneity of variance (Levene’s test) and one-way ANOVA, with Fisher LSD post hoc test when *p* < 0.05. Data are presented as mean ± standard error of the mean (SEM).

## Results

### Dietary LNA Increased Tissue Arachidonic Acid and Endocannabinoid Levels

**Fig. 1:**
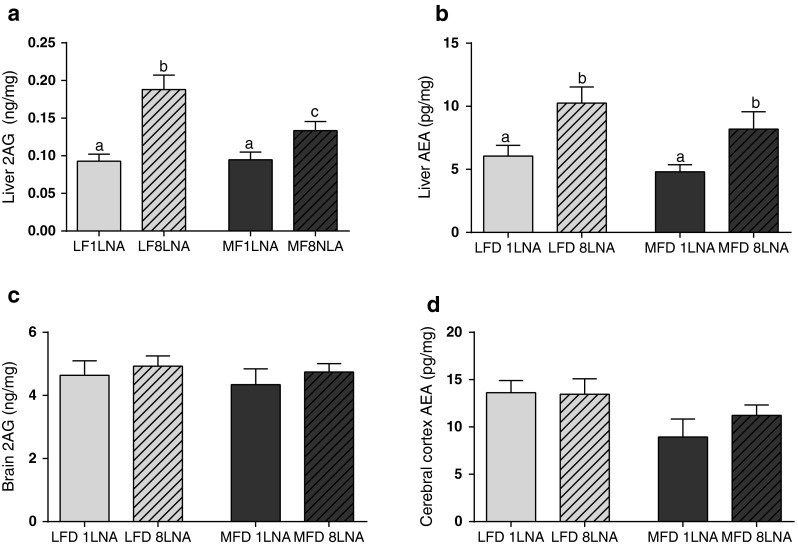
Selective elevation of dietary LNA elevates liver endocannabinoids in mice fed low fat diets (LFD) of 12.5 en% fat (*light gray*) and medium fat diets (MFD) of 35 en% fat (*dark gray*). Increasing dietary LNA from 1 en% (*open bars*) to 8 en% (*hatched bars*) in both LFD and MFD elevated (**a**) liver 2-AG and (**b**) AEA, an increase that was not observed in the cerebral cortex (**c**, **d**). *Different superscript letters* indicate a statistical significance *p* < 0.05 by ANOVA, *n* = 8–9

Diets containing 8 en%, reflecting current US intake, resulted in significantly higher amounts of LNA and ARA in RBC–PL (Table 2), liver–PL (Table 3), and eWAT (Table 4) compared to diets containing 1 en% LNA. Consequently, elevating LNA from 1 to 8 en% in both LFD and MFD significantly increased 2-AG and AEA in liver (Fig. 1a, b). The brain is less influenced by dietary manipulation and we found no differences in endocannabinoid levels in the cerebral cortex (Fig. 1c, d). Elevating dietary LNA from 1 to 8 en% significantly reduced EPA–PL in both RBC (Table 2) and liver (Table 3). The concentration of DHA–PL was increased in RBC of mice fed LFD and MFD containing 1 en% LNA (Table 2), but only in livers of mice fed LFD 1 en% (Table 3). Thus, increasing dietary LNA from 1 to 8 en% decreased the n-3 index in RBC-PL from 9 to 5 (Table 2). Mice fed 8 en% LNA had significantly higher EPA and DHA in eWAT than mice fed 1 en% LNA in both LFD and MFD (Table 4).

**Table 2:** Fatty acid profile in RBC-PL [μg FA/g RBC, (w.w.)]

FA (μg/g)	Low fat (12 en% fat)		Medium fat (35 en% fat)	
	1 en% LNA	8 en% LNA	1 en% LNA	8 en% LNA
16:0	529 ± 19^a^	590 ± 11^b^	578 ± 21^ab^	585 ± 18^b^
18:0	172 ± 7^a^	246 ± 8^b^	228 ± 9^b^	271 ± 9^c^
Sum SFA	718 ± 26^a^	858 ± 19^b^	840 ± 30^b^	887 ± 28^b^
18:1n9	395 ± 13^a^	204 ± 5^b^	375 ± 12^a^	347 ± 7^c^
Sum MUFA	518 ± 16^a^	287 ± 7^b^	503 ± 16^a^	323 ± 9^c^
18:2n-6	111 ± 6^a^	272 ± 8^b^	158 ± 6^c^	291 ± 11^b^
20:4n-6	293 ± 11^a^	407 ± 8^b^	324 ± 15^a^	404 ± 12^b^
22:5n-6	11.2 ± 0.4^a^	16.3 ± 0.4^b^	12.4 ± 0.6^a^	16.9 ± 0.8^b^
Sum n-6	497 ± 17^a^	775 ± 15^b^	557 ± 20^c^	791 ± 29^b^
18:3n-3	3.3 ± 0.4^a^	2.8 ± 0.1^a^	4.7 ± 0.3^b^	2.1 ± 0.1^c^
20:5n-3	19.6 ± 1.3^a^	7.4 ± 0.4^b^	19.8 ± 0.7^a^	8.9 ± 0.4^b^
22:5n-3	14.6 ± 0.9^a^	18.1 ± 0.6^b^	18.6 ± 0.9^b^	18.2 ± 0.7^b^
22:6n-3	179 ± 7^ab^	164 ± 4^ac^	180 ± 7^b^	158 ± 3^c^
Sum n-3	220 ± 9^a^	193 ± 5^b^	228 ± 8^a^	188 ± 4^b^
n-6/n-3 ratio	2.1 ± 0.0^a^	4.0 ± 0.1^b^	2.5 ± 0.0^c^	4.2 ± 0.1^d^
n-6 HUFA (%)	62 ± 0^a^	73 ± 0^b^	64 ± 1^c^	73 ± 0^b^
n-3 HUFA (%)	38 ± 0^a^	27 ± 0^b^	34 ± 1^c^	27 ± 0^b^
Omega-3 Index	9 ± 0^a^	5 ± 0^b^	8 ± 0^c^	5 ± 0^b^

Different superscript letters indicate significant statistical differences, Mann–Whitney *p* < 0.01, n = 8–9. Omega-3 Index is calculated from (n-3 HUFA × 0.32) − 3.5 [28, 59]). Tissue levels of n-6 HUFA is in good concurrency with estimated n-6 HUFA values in Table 1

*LNA* linoleic acid, *SFA* saturated fatty acids, *MUFA* monounsaturated fatty acids, *HUFA* highly unsaturated fatty acids

**Table 3:** Fatty acid profile in liver-PL [mg FA/g liver (w.w.)]

FA (mg/g)	Low fat (12 en% fat)		Medium fat (35 en% fat)	
	1 en% LNA	8 en% LNA	1 en% LNA	8 en% LNA
16:0	3.6 ± 0.2	3.4 ± 0.3	3.4 ± 3.2	3.5 ± 0.1
18:0	2.0 ± 0.1^a^	2.3 ± 01^ab^	2.1 ± 0.1^a^	2.5 ± 0.1^b^
Sum SFA	5.8 ± 0.3	5.8 ± 0.4	5.6 ± 0.3	6.2 ± 0.2
18:1n-9	2.9 ± 0.1^a^	1.2 ± 0.1^b^	2.2 ± 0.1^c^	1.3 ± 0.1^b^
Sum MUFA	4.5 ± 0.1^a^	2.0 ± 0.2^b^	3.6 ± 0.2^c^	2.1 ± 0.11^b^
18:2n-6	1.2 ± 0.1^a^	2.3 ± 0.2^b^	1.0 ± 0.1^a^	2.2 ± 0.1^b^
20:4n-6	1.9 ± 0.1^a^	3.1 ± 0.2^b^	1.8 ± 0.1^a^	3.2 ± 0.2^b^
Sum n-6	3.5 ± 0.2^a^	5.9 ± 0.5^b^	3.6 ± 0.2^a^	6.0 ± 0.3^b^
20:5n-3	0.14 ± 0.02^a^	0.09 ± 0.01^b^	0.12 ± 0.01^ab^	0.10 ± 0.00^b^
22:5n-3	0.07 ± 0.02^a^	0.09 ± 0.01^ab^	0.09 ± 0.01^ab^	0.10 ± 0.00^b^
22:6n-3	2.4 ± 0.1^a^	1.9 ± 0.3^b^	2.0 ± 0.1^b^	1.9 ± 0.1^b^
Sum n-3	2.6 ± 0.2^a^	2.1 ± 0.2^b^	2.2 ± 0.1^ab^	2.1 ± 0.1^b^
n-6/n-3 ratio	1.4 ± 0.01^a^	2.8 ± 0.1^b^	1.5 ± 0.1^a^	2.9 ± 0.1^b^
n-6 HUFA (%)	47 ± 1^a^	62 ± 1^b^	50 ± 1^a^	64 ± 1^b^

Different superscript letters indicate significant statistical differences, Mann–Whitney *p* < 0.01, *n* = 8–9

*LNA* linoleic acid, *SFA* saturated fatty acids, *MUFA* monounsaturated fatty acids, *HUFA* highly unsaturated fatty acids

**Table 4:** Fatty acid profile in neutral lipids of white epididymal adipose tissue [mg FA/g eWAT (w.w.)]

FA (mg/g)	Low fat (12 en% fat)		Medium fat (35 en% fat)	
	1 en% LNA	8 en% LNA	1 en% LNA	8 en% LNA
16:0	133 ± 3^ac^	158 ± 3^b^	149 ± 3^bc^	140 ± 5^c^
18:0	8.3 ± 0.3^a^	12.5 ± 0.4^b^	8.3 ± 0.2^a^	10.5 ± 0.5^c^
Sum SFA	163 ± 4^a^	189 ± 4^b^	297 ± 4^c^	268 ± 13^d^
18:1n9	489 ± 5^a^	236 ± 5^b^	360 ± 4^c^	301 ± 6^d^
Sum MUFA	614 ± 6^a^	333 ± 6^b^	497 ± 4^c^	393 ± 9^d^
18:2n-6	57 ± 3^a^	308 ± 3^b^	42 ± 3^c^	187 ± 4^d^
20:4n-6	1.1 ± 0.0^a^	4.0 ± 0.2^b^	0.8 ± 0.0^a^	2.6 ± 0.1^c^
22:5n-6	0.1 ± 0.0^a^	0.4 ± 0.1^b^	0.0 ± 0.0^a^	0.2 ± 0.1^c^
Sum n-6	59 ± 3^a^	317 ± 3^b^	43 ± 3^c^	192 ± 4^d^
18:3n-3	5.8 ± 0.2^a^	19.1 ± 0.4^b^	4.4 ± 0.1^c^	12.1 ± 0.2^d^
20:5n-3	0.1 ± 0.0^a^	0.4 ± 0.0^b^	0.1 ± 0.0^a^	0.3 ± 0.0^c^
22:5n-3	0.2 ± 0.0^a^	0.7 ± 0.1^b^	0.2 ± 0.0^a^	0.5 ± 0.0^c^
22:6n-3	1.5 ± 0.1^a^	2.5 ± 0.1^b^	1.1 ± 0.1^c^	1.7 ± 0.1^a^
Sum n-3	8.9 ± 0.2^a^	23.5 ± 0.5^b^	7.2 ± 0.1^c^	14.8 ± 0.3^d^
n-6/n-3 ratio	6.7 ± 0.3^a^	13.6 ± 0.3^b^	6.0 ± 0.4^a^	13.1 ± 0.3^b^
n-6 HUFA (%)	49 ± 1^a^	69 ± 0^b^	46 ± 2^a^	67 ± 1^b^

Different superscript letters indicate significant statistical differences, Mann–Whitney *p* < 0.01, *n* = 8–9

*LNA* linoleic acid, *SFA* saturated fatty acids, *MUFA* monounsaturated fatty acids, *HUFA* highly unsaturated fatty acids

### Dietary LNA Increased Weight Gain and Body Weight in Low Fat Diets

**Fig. 2:**
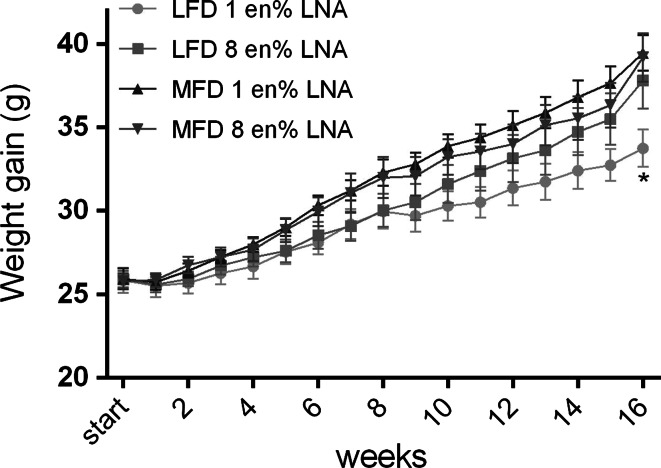
Weekly weight gain. After 16 weeks of feeding the LFD of 8 en% LNA resulted in similar weight gain as the MFD, significantly higher than an LFD of 1 en% LNA

Food intake was not affected by the dietary treatments (Table 5). As expected, mice fed MFDs gained more body weight and tended to accumulate more adipose tissue than mice fed an LFD when dietary LNA was kept low at 1 en% (Fig. 2, Table 5). Of note, when dietary LNA was elevated from 1 to 8 en% in the low fat diet, feed efficiency increased resulting in a similar weight gain and body weight as the MFDs (Fig. 2, Table 5). Muscle weight did not differ between the groups (data not shown), indicating that differences in body weight was due to accumulation of adipose tissue and not lean mass.

**Table 5:** Physical and biochemical parameters

Parameter	Low fat (12 en% fat)		Medium fat (35 en% fat)	
	1 en% LNA	8 en% LNA	1 en% LNA	8 en% LNA
Total food intake (g/112 days)	357 ± 8^a^	361 ± 10^a^	322 ± 7^b^	323 ± 5^b^
Total caloric intake (kcal)	1,398 ± 33	1,443 ± 39	1,387 ± 28	1,361 ± 19
Feed efficiency (BW gain/mcal)	5.6 ± 0.5^a^	8.1 ± 0.8^b^	9.7 ± 0.5^b^	9.8 ± 0.8^b^
Body weight at start (g)	25.8 ± 0.8	25.9 ± 0.5	25.9 ± 0.6	25.8 ± 0.5
Final body weight (g)	33.7 ± 1.1^a^	37.8 ± 1.7^b^	39.4 ± 1.1^b^	39.2 ± 1.5^b^
Weight gain (g)	7.9 ± 0.8^a^	11.9 ± 1.4^b^	13.5 ± 0.9^b^	13.4 ± 1.2^b^
WAT (g)	1.8 ± 0.3^a^	2.4 ± 0.3^a,b^	2.4 ± 0.3^a,b^	2.8 ± 0.3^b^
Adiposity Index (%)	5.9 ± 0.9^a^	7.5 ± 0.7^ab^	7.4 ± 0.9^ab^	8.6 ± 0.7^b^
Leptin (ng/mL)	4.4 ± 1.3^a^	11.4 ± 2.5^b^	6.1 ± 0.8^a^	13.4 ± 1.3^b^
Adiponectin (μg/mL/g WAT)	2.0 ± 0.2^a^	2.9 ± 0.1^b^	1.6 ± 0.1^c^	2.8 ± 0.1^b^
Insulin (μg/L)	2.4 ± 0.5	3.2 ± 0.5	3.7 ± 0.5	2.9 ± 0.6

Different superscript letters indicate significant statistical differences, ANOVA *p* < 0.05, *n* = 9, hormones; *n* = 4–7 (difference in *n* due loss of samples when kits were re-run with proper dilution). Adiposity index [(subcutaneous + retroperitoneal + inguinal fat pads)/eviscerated body weight × 100]

### Dietary LNA Elevated Plasma Leptin Levels

The LFD and MFD containing 8 en% LNA significantly elevated plasma leptin concentrations compared to 1 en% LNA diets (Table 5). Mice fed LFDs of 1 and 8 en% LNA had similar plasma adiponectin levels per gram fat as mice fed 8 en% LNA in the MFD and significantly higher body fat levels than mice fed 1 en% in the MFD (Table 5). Plasma insulin concentrations were not affected by the dietary treatments (Table 5).

### Dietary LNA Increased Adipose Accumulation and Adipocyte Size

**Fig. 3:**
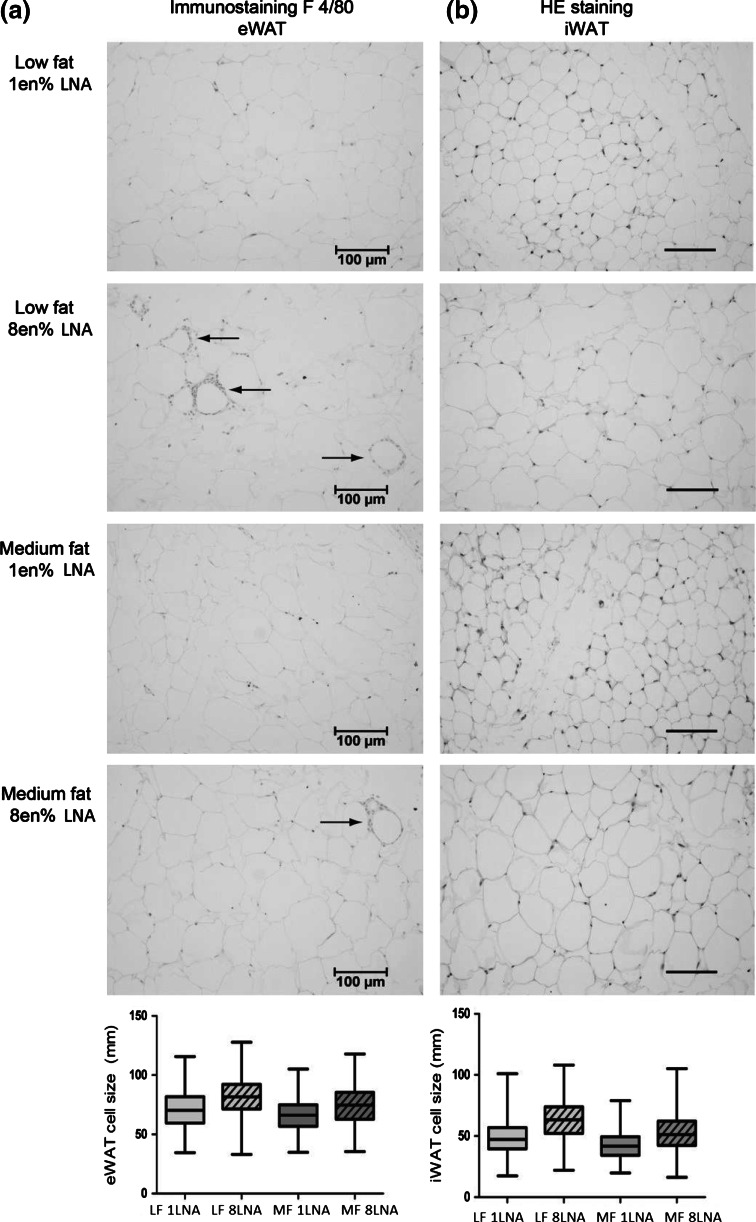
Dietary LNA increase macrophage infiltration and cell size in adipose tissue. (**a**) Immunostaining with F4/80 showed more crown-like structures indicating macrophage infiltration (indicated by *arrows*) in eWAT of mice fed 8 en% LNA compared to mice fed 1 en% LNA. (**b**) HE staining of iWAT. Mice fed 8 en% LNA displayed larger adipocyte cell size in (**a**) eWAT and (**b**) iWAT in both low fat (LF) and medium fat diets (MF) compared to mice fed 1 en% LNA. Data are presented as min to max, line at median, *n* = 2. *Scale bar* 100 μm

The accumulation of white adipose tissue (WAT) and the adiposity index [(subcutaneous + retroperitoneal + inguinal fat pads)/eviscerated body weight × 100] was similar in mice fed 8 en% LNA in an LFD and mice fed the MFDs (Table 5). Mice fed 1 en% LNA in the LFD had significantly less adipose tissue and lower adiposity index than mice fed 8 en% LNA in a MFD (Table 5). We performed immunohistochemical analysis of adipose tissue by staining for the MI macrophage marker F4/80. We only found macrophage infiltration in eWAT of mice fed 8 en% LNA diets inducing F4/80 positive macrophages forming crown-like structures around adipocytes (Fig. 3a). No macrophage infiltration was found in iWAT (data not shown). These results indicate that the composition of dietary fat is substantially more important than the total amount of fat in determining adipose tissue accumulation and macrophage infiltration (Fig. 3a, b).

## Discussion

We have previously shown that modeling the increase in human consumption of dietary LNA from 1 to 8 en% LNA during the last century [23] caused excessive endocannabinoid levels and also induces obesity in mice fed diets of 35 and 60 en% fat [14]. To our knowledge, we demonstrate here for the first time that an LFD can be made obesogenic by inclusion of 8 en% LNA, subsequently stimulating excessive endocannabinoid activity in the liver and weight gain. In the present study we report that isocalorically increasing dietary LNA from 1 to 8 en% elevated tissue levels ARA–PL, liver 2-AG and AEA regardless of changes in total dietary fat, and increased feed efficiency, caused higher weight gain and elevated plasma leptin in mice fed the LFD. We also observed a tendency of increased cell size and macrophage infiltration in eWAT in mice fed 8 en% LNA.

The percent of n-6 in HUFA can be used as an indicator of disease risk as it models the relative amounts of tissue ARA–PL available as precursors for eicosanoid derivatives of ARA [28]. We used the Lands equation [25] to predict tissue ARA–PL composition as the % n-6 HUFA from dietary intakes of n-6 and n-3 PUFA to model availability of endocannabinoid precursors with good concurrence between calculated and experimental values. Here we modeled the increase in US dietary intakes of LNA from 1 to 8 en% [23] in our animal diets, and showed in mice, that elevating dietary LNA to 8 en% increased ARA–PL in liver, RBC and adipose tissue to levels currently found in Americans [29], and induced elevations in liver 2-AG and AEA. Elevating endocannabinoid levels by dietary LNA to alter the availability of ARA in the phospholipid precursor pool is consistent with several studies where dietary fatty acids alter endocannabinoid levels [30–36], and our previous work where we selectively altered LNA and raised dietary omega-3 HUFA both in mice and salmon [14, 15]. We did not observe any differences in food intake or endocannabinoid levels in the cerebral cortex, suggesting that the effects of increased endocannabinoid levels were caused by effects in peripheral tissue. The adult brain is less susceptible to dietary manipulations than the young brain [37, 38]. The lower sensitivity of dietary LNA on brain endocannabinoid levels compared to peripheral tissues confirm that brain lipids levels are less influenced by changes in dietary fatty acids and that this may be reflected also in the endocannabinoids [39, 40].

Dietary LNA of 8 en%, independent of dietary fat content, significantly elevated plasma leptin levels compared to 1 en% LNA. Plasma adiponectin levels were less affected by dietary LNA, suggesting that leptin is a more sensitive marker of early obesity development. Mice fed an LFD had similar plasma concentrations of adiponectin per gram adipose tissue despite higher WAT and adiposity in mice fed 8 en% LNA compared to 1 en% LNA. This result suggests that mice fed a high LNA content secrete less adiponectin per gram fat, in line with the obesity-prone phenotype of the 8 en% LNA diet.

We find that the LFD containing 8 en% LNA significantly increased feed efficiency compared to the 1 en% LNA diet causing the animals to gain more weight per calorie consumed. The increased feed efficiency and weight gain in mice fed 8 en% LNA in the LFD resulted in a similar body weight as mice fed 35 en% fat, significantly higher than mice fed 1 en% LNA in an LFD. Dietary LNA of 1 en% has been found to reverse the obesogenic properties of a high fat diet [14]. In line with previous studies, our findings in the LFD support the notion that weight gain involves mechanisms that depend more on the composition of dietary fat than the total amount of fat in the diet [12, 30, 41–43]. Increased intake of LNA has been linked to increased prevalence of obesity [26, 41] and the primary dietary sources of LNA; soybean oil, poultry and shortening were positively correlated to increasing rates of obesity occurring in the US during the 20th century [14]. Replacing saturated fats with LNA-rich vegetable oil increased body weight in veterans [44] and ARA levels in adipose tissue were positively associated with BMI in children [45]. In animals, vegetable oils rich in LNA (usually soybean oil; 55 % LNA, and safflower oil; 75 % LNA) elevated food intake [14, 15, 46], induced weight gain [14, 15, 30, 41, 42, 47] and increased lipogenic enzyme activity in the liver [10, 48]. Our findings imply that low fat diets could be made more effective in reducing adiposity if LNA were lowered to near 1 en%. Indeed, total dietary fat intake may not need to be lowered if LNA is selectively lowered.

In contrast to what we previously reported [14], 1 en% LNA in the MFD did not prevent weight gain and adiposity compared to MFD 8 en% LNA. In the present study animals were fed the experimental diets from 6 weeks of age, whereas in our previous study animals were exposed to the dietary treatments from the last week of pregnancy to 17 weeks of age [14]. D’Asti et al. [49] demonstrated how quantity and quality of maternal dietary fat during the perinatal period directly influences neonatal metabolism, fatty acid composition in phospholipids and sensitivity to endocannabinoid system manipulation. Massiera et al. [50] found a gradual transgenerational increase in adiposity in mice fed a “western-like” diet of 35 en% fat containing 18 en% LA for four generations. Thus the adipogenic effect of LNA appears to be higher when prenatally exposed [14]. Additionally, in the present study, mice were fed a standard NIH #31M diet based on soybean oil prior to arrival at our facility (Taconic, Denmark). Calculations by the Lands equation [25] show that the NIH #31M diet results in 72 % n-6 HUFA in tissue phospholipids, identical to our diets containing 8 en% LNA. Metabolic changes such as induced lipogenesis and deposition of lipid droplets in response to increased endocannabinoid activity have been shown to occur before onset of obesity [10, 12, 51]. It is possible that an already elevated endocannabinoid activity present in the animals upon arrival to our facility may have overridden the anti-adipogenic properties of the 1 en% LNA diet, when the MFD contained abundant carbohydrates [42]. The latter might be of importance as it has been demonstrated that the obesogenic potential of diets rich in both n-6 and n-3 PUFA is elevated by increasing the levels of dietary carbohydrates, and thereby raising the insulin/glucagon ratio translated into reduced cAMP signaling [42, 52]. cAMP signaling has been demonstrated to play a pivotal role in controlling the production of both pro- and anti-adipogenic prostaglandins both in vivo and in vitro [42]. Collectively these results illustrate the importance of the type of fat and carbohydrates in the background diet, and how these can influence the obesogenic properties of LNA. Mice fed an LFD of 8 en% LNA started to gain considerably more weight than mice fed 1 en% LNA after 8 weeks of feeding. Thus a longer feeding period may be necessary to wash out excessive tissue ARA caused by the NIH #31M diet, before a beneficial effect of a low LNA diet (1 en%) may occur. Although body weight did not differ, elevated liver 2-AG and AEA, larger adipocytes, higher macrophage accumulation in WAT and elevated plasma leptin levels in mice fed 8 en% may suggest a more obesity prone phenotype and a higher risk of developing obesity and metabolic complications associated with obesity compared to mice fed same amount of fat but with only 1 en% LNA.

One of the major limitations when working with dietary fat is to relate the observed differences to one fatty acid. There are no inert oils which can be added to a diet to target one single fatty acid to be different in a diet. We did extensive analyses of various fats and oils available on the market to establish the fatty acid profile of the products and carefully mixed several oils to isolate LNA as controlled variable via isocaloric replacement of saturated fat (in the medium fat diet). It was not possible to obtain similar content of MUFA in the low fat diets since the low fat diet contained 8 en% fat as LA of the total of 12.5 en% fat. Although our diets contained different amounts of SFA and MUFA, SFA were covariable in the LFD and MUFA were covariable in the MFD, thus strengthening our conclusion that LNA is the major dietary fatty acid affecting endocannabinoid levels and induces weight gain. If there were other fatty acids (MUFA or SFA) that strongly influenced endocannabinoid levels and adiposity we would expect the differences between the dietary treatments to be less distinct. Another limitation with our study is the absence of endocannabinoid measurements in adipose tissue, making it difficult to conclude if the observed elevation of endocannabinoid levels in the liver is a consequence rather than the cause of obesity. In order to establish a cause–effect relationship between a diet induced elevation in endocannabinoid levels and the observed obese phenotype in mice fed the LFD of 8 en% LNA a follow-up study should be carried out using a CB1 antagonist.

Reducing dietary LNA from 8 to 1 en% with a concomitant decrease in ALA from 1 to 0.3 en% significantly increased EPA–PL concentrations in RBC (three-fold) and liver, increasing the n-3 index from 5 to 8 and 9 in MFD and LFD respectively. We have previously reported that reducing LNA from 8 to 1 en% increased EPA and reduced ARA in liver and RBC similarly to supplementing an 8 en% LNA diet with 1 en% EPA + DHA [14]. Consistent with previous reports [53–58], our results indicate that the elongation and desaturation of ALA to EPA and DHA are considerably more effective when dietary LNA is 1 en%. Hence to improve tissue EPA and DHA concentrations, reduce ARA–PL and consequently decrease endocannabinoid production, emphasis should be on lowering dietary LNA in addition to dietary supplementation with EPA and DHA. In conclusion, a dietary approach by reducing substrate availability for endocannabinoid synthesis provides a safe and preventative alternative to decrease hyperactivity of the endocannabinoid system, and consequently decrease or prevent obesity.

## Abbreviations

2-AG: Arachidonoylglycerol
ARA: Arachidonic acid
AEA: Anandamide
CB: Cannabinoid receptor
DHA: Docosahexaenoic acid
en%: Percent of energy
EPA: Eicosapentaenoic acid
eWAT: Epididymal white adipose tissue
HUFA: Highly unsaturated fatty acid(s) (fatty acids of >20 carbon length)
iWAT: Inguinal white adipose tissue
LFD: Low fat diet
LNA: Linoleic acid
MFD: Medium fat diet
MUFA: Monounsaturated fatty acid(s)
PL: Phospholipids
RBC: Red blood cells
SFA: Saturated fatty acid(s)
